# High Expression of GOLPH3 in Esophageal Squamous Cell Carcinoma Correlates with Poor Prognosis

**DOI:** 10.1371/journal.pone.0045622

**Published:** 2012-10-02

**Authors:** Jian-Hua Wang, Xiu-Ting Chen, Zhe-Sheng Wen, Min Zheng, Jian-Ming Deng, Ming-Zhi Wang, Huan-Xin Lin, Kun Chen, Jun Li, Jing-Ping Yun, Rong-Zhen Luo, Li-Bing Song

**Affiliations:** 1 Department of Chest, Second People's Hospital of Guangdong Province, Guangzhou, Guangdong, P. R. China; 2 State Key Laboratory of Oncology in South China, Sun Yat-sen University Cancer Center, Guangzhou, Guangdong, P. R. China; 3 Department of Chest, Sun Yat-sen University Cancer Center, Guangzhou, Guangdong, P. R. China; 4 Department of Gynecology, Sun Yat-sen University Cancer Center, Guangzhou, Guangdong, P. R. China; 5 Department of Radiotherapy, Sun Yat-sen University Cancer Center, Guangzhou, Guangdong, P. R. China; 6 Department of Pathology, Sun Yat-sen University Cancer Center, Guangzhou, Guangdong, P. R. China; 7 Neurosurgery, First Affiliated Hospital of Sun Yat-sen University, Guangzhou, Guangdong, P. R. China; 8 Biochemical Institute, Sun Yat-sen University, Guangzhou, Guangdong, P. R. China; The University of Hong Kong, China

## Abstract

**Background:**

Whether the expression of Golgi phosphoprotein 3 (GOLPH3) correlates with esophageal cancer tumorigenesis is currently unclear. The aim of this study was to examine GOLPH3 expression in patients with esophageal squamous cell cancer (ESCC) and explore its clinical significance.

**Methods:**

Differences in the expression of GOLPH3 at the mRNA and protein level were examined via quantitative reverse transcriptase PCR and western blotting, respectively. GOLPH3 expression levels in ESCC tissue were determined through immunohistochemistry, and were compared in accordance with specific clinicopathological features of the patients and tissue specimens. Factors associated with patient survival were also analyzed.

**Results:**

A notably higher level of GOLPH3 expression was found in ESCC cell lines and tissues at both mRNA and protein levels. High expression of GOLPH3 in ESCC patients was positively associated with clinical stage, TNM classification, histological differentiation and vital status (all *P*<0.0001). Expression of GOLPH3 was found to be an independent prognostic factor in ESCC patients. ESCC patients expressing high levels of GOLPH3 exhibited a substantially lower 5-year overall survival than GOLPH3-negative patients. Furthermore, a significant correlation between high GOLPH3 expression and shorter overall survival time was found in different subgroups of ESCC patients stratified by the clinical stage, T classification, and lymph node metastasis.

**Conclusions:**

Experiments demonstrated potential involvement of GOLPH3 in the development, differentiation, and tumorigenesis of ESCC, and concludes the possibility of its use as a diagnostic and prognostic marker in patients with ESCC.

## Introduction

Esophageal cancer ranks as the ninth most common malignancy and sixth most frequent cause of cancer death worldwide, with occurrence rates varying greatly by geographic location [1]. According to a recent survey, China, a region with relatively high incidence, reports 167,200 cases of esophageal cancer out of a global total of approximately 310,400 each year [2]. Esophageal cancer distributes a general poor prognosis due to lack of a singular effective clinical method for early diagnosis. The overall 5-year survival rate for esophageal cancer is approximately 15% [3].

The etiology of esophageal cancer is a complex process that involves cumulative mutations in multiple genes, but its exact pathogenesis is still unclear. Thus, the identification of effective therapeutic, diagnostic, and/or prognostic marker genes for esophageal cancer is a critically imminent issue. Previous reports have shown that genetic changes frequently associated with the development of esophageal cancer include the *p53* mutation, inactivation of *p16*, cyclin D1 amplification, and overexpression of *c-Myc* or *EGFR* [4]–[5].

Recently, Golgi phosphoprotein 3 (*GOLPH3*) has been demonstrated as a novel oncogene involved in the development of cancer of the lung, ovary, breast, colon and prostate, as well as melanoma, rhabdomyosarcoma, and glioma [6]–[9]. GOLPH3 was initially detected as a phosphorylated protein localized to the Golgi apparatus [10]–[11]. Recent functional, cell biological, and biochemical analyses have shown that GOLPH3 can induce cell transformation and tumor growth by enhancing the activity of the mammalian target of rapamycin complex (mTORC) [6], [10]–[13]. GOLPH3's relation with esophageal cancer, however, remains unclear.

The objective of this study was to investigate the expression of GOLPH3 in esophageal squamous cell cancer (ESCC) and further explore its clinical significance. In this study, quantitative reverse transcriptase PCR (qRT-PCR), Western blot analysis, and immunohistochemistry methods were used to examine GOLPH3 mRNA and protein expression. Correlation of GOLPH3 expression with clinicopathological features specific to ESCCs was also assessed.

## Materials and Methods

### Patient information and tissue specimens

Patients taken for this study were diagnosed with ESCC from 2001 to 2003 at the Sun Yat-sen University Cancer Center, and underwent esophageal cancer resection, prior to the administration of chemotherapy (Table 1). ESCC and carcinoma-adjacent tissue were obtained from resected tumors and adjacent non-tumorous esophageal tissues, respectively, and confirmed by pathological review. All samples were obtained from the Tissue Bank of the Sun Yat-sen University Cancer Center and coded anonymously in accordance with local ethical guidelines (as stipulated by the Declaration of Helsinki). Written informed consent was obtained from patients, and protocol was approved by the Review Board of Sun Yat-sen University Cancer Center (approval number: YP2011024). ESCC specimens were staged in accordance with American Joint Cancer Committee/Union Internationale Contre le Cancer (UICC/AJCC) classification guidelines. The grading and histopathology subtyping of ESCC specimens was based on WHO criteria. Eight ESCC biopsy samples and their adjacent matching noncancerous esophageal tissues were frozen in liquid nitrogen and stored.

**Table 1:** Clinicopathological characteristics of patient samples and expression of GOLPH3 in ESCC.

	Number of cases (%)
**Gender**	
Male	118(76.1)
Female	37(23.9)
**Age (years)**	
≤57	83(53.5)
>57	72(46.5)
**Clinical stage**	
I	9(5.8)
IIA	64(41.3)
IIB	14(9.0)
III	54(34.8)
IV	14(9.0)
**T classification**	
T1	11(7.1)
T2	43(27.7)
T3	91(58.7)
T4	10(6.5)
**N classification**N0	
N0	78(50.3)
N1	74(47.7)
N2	3(1.9)
**M classification**	
M0	141(91.0)
M1	14(9.0)
**Differentiation**	
Well	47(30.3)
Moderate	57(36.8)
Poor	51(32.9)
**Vital status (at follow-up)**	
Alive	64(41.3)
Death (tumor-related)	89(57.4)
Death (tumor-unrelated)	2(1.3)
**Expression of GOLPH3**	
Low expression	79(51.0)
High expression	76(49.0)
**Location**	
Upper	17(11.0)
Middle	88(56.8)
Lower	50(32.3)
**Therapy**	
Surgery only	132(85.2)
surgery + CT or RT or CRT	23(14.8)
**Completeness of surgical resectionon**	
Yes	149(96.1)
No	6(3.9)
**Complication**	
Yes	35(22.6)
No	120(77.4)

Abbreviations: **CT**, chemotherapy; **RT**, radiotherapy; **CRT**, combination of CT and RT (chemoradiotherapy).

### qRT-PCR

Total RNA was extracted using Trizol reagent (Invitrogen Life Technologies, Ontario, Canada). The qPCR primers to amplify GOLPH3 were designed using the qPrimerDepot website (http://primerdepot.nci.nih.gov/). GOLPH3 primer patterns are as follows: 5′- GGGCGACTCCAAGGAAAC -3′ (forward) and 5′- CAGCCACGTAATCCAGATGAT -3′ (reverse), and glyceraldehyde 3-phosphate dehydrogenase (GAPDH) primers included: 5′- ATTCCACCCATGGCAAATTC -3′, (forward) and 5′- ATTCCACCCATGGCAAATTC -3′ (reverse). qRT-PCR was carried out using the FastStart Universal SYBR Green Master (ROX; Roche, Toronto, ON, Canada) on the Bio-Rad CFX96 qRT-PCR detection system (Applied Biosystems Inc., Foster City, CA, USA). The CFX Manager software was used to calculate a threshold cycle (Ct) value for GAPDH and GOLPH3 during the log phase of each cycle. Expression data were normalized to the geometric mean of the housekeeping gene *GAPDH* to control the variability in expression levels, and then analyzed using the 2^−ΔΔct^ method, where ΔΔCt = ΔCt_GOLPH3_ – ΔCt_GAPDH_. To minimize experimental variability, each sample was tested in triplicate and the mean femtogram expression level was calculated.

### Western blotting analysis

For each sample, 40 µg of total protein was taken, according to the method described in Planchamp *et al* [14]. The mouse polyclonal antibody to GOLPH3 (1∶1000) (ab69171; Abcam plc, Cambridge, UK) and the goat anti-mouse secondary antibody (1∶2000) (Sigma, St. Louis, MO, USA) were used in the assay. Bound antibodies were visualized using the ECL system (Amersham Pharmacia Biotech, Dübendorf, Switzerland).

### Immunohistochemistry

Paraffin-embedded tissues were analyzed using immunohistochemical staining as described by Zheng *et al* [15], in which the anti-GOLPH3 antibody was a mouse polyclonal antibody (1∶50) (ab69171; Abcam plc, Cambridge, UK). Control samples were stained in parallel, but were not incubated with either primary or secondary antibodies. The degree of immunostaining of formalin-fixed, paraffin-embedded sections was reviewed and independently scored by two pathologists previously uninformed of the histopathological features and patient data of the samples. Scores were determined by combining the proportion of positively stained tumor cells and the intensity of staining. Scores given by the two pathologists were combined into a mean score for further comparative evaluation. Tumor cell proportions were scored as follows: 0 (no positive tumor cells); 1 (<10% positive tumor cells); 2 (10–35% positive tumor cells); 3 (35–75% positive tumor cells) and 4 (>75% positive tumor cells). Staining intensity was graded according to the following standard: 1 (no staining); 2 (weak staining  =  light yellow); 3 (moderate staining  =  yellow brown) and 4 (strong staining  =  brown). The staining index (SI) was calculated as the product of the staining intensity score and the proportion of positive tumor cells. Using this method of assessment, we evaluated GOLPH3 expression in benign esophageal epithelia and malignant lesions by determining the SI, with scores of 0, 1, 2, 3, 4, 6, 8, 9, 12, or 16 (Figures S1, S2). Table of IHC score tabulates the number and distribution of cases according to the immunohistochemistry score (Table S1). Mean score is 6.86, median value is 8. The cutoff value for high and low expression was determined on the basis of a heterogeneity value measured through log-rank statistical analysis with respect to overall survival. An SI score ≥8 defined tumors with high GOLPH3 expression, and an SI score ≤6 indicated low expression.

Results of immunohistochemical (IHC) staining of tumor and normal tissues were quantitative analyzed with the AxioVision Rel.4.6 computerized image analysis system assisted with the automatic measurement program (Carl Zeiss, Oberkochen, Germany). Briefly, the stained sections were evaluated at 200x magnification, and 10 representative staining fields of each section were analyzed to obtain the mean optical density (MOD), which represents the strength of staining signals as measured per positive pixels. The MOD data were statistically analyzed using t-tests to compare the average MOD difference between different groups of tissues.

### Statistical analysis

Pearson's Chi-square test and Spearman correlation analysis were also applied in studying the relationship between GOLPH3 expression and patient characteristics including age, gender, tumor stage, TNM classification, histological differentiation, and vital status. Survival curves for both GOLPH3-high and GOLPH3-low expression patients were plotted using the Kaplan–Meier method, and statistical differences were compared using a log-rank test. Univariable and multivariable survival analysis was performed using the Cox regression analysis. A *P*-value of less than 0.05 was considered statistically significant. All statistical analyses were performed using the SPSS 13.0 (IBM, Armonk, NY, USA) statistical software package.

## Results

### Increased expression of GOLPH3 in ESCC at both mRNA and protein levels

**Figure 1:**
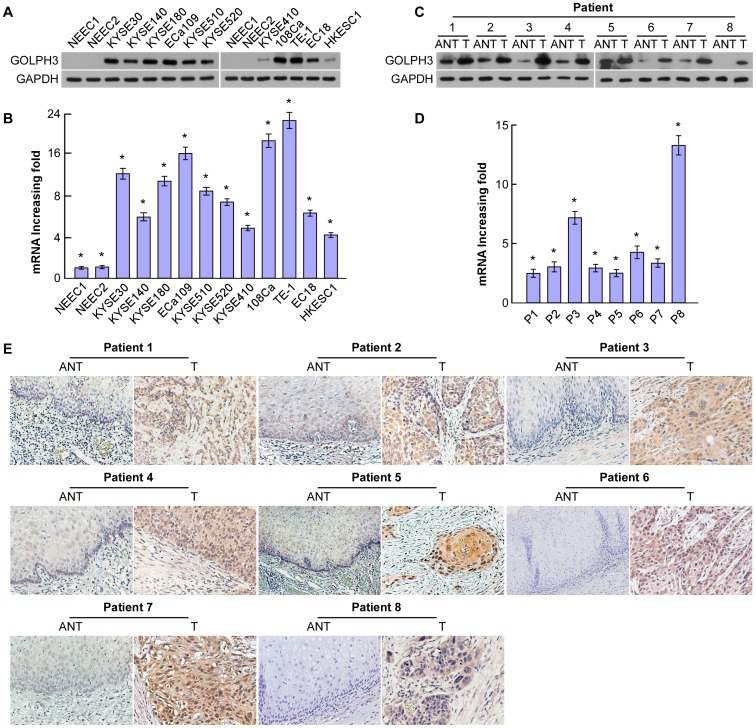
Increased GOLPH3 expression detected in ESCC cell lines and tissues by qRT-PCR and western blotting. GOLPH3 mRNA and protein levels in 11 ESCC cell lines (KYSE-30, KYSE-140, KYSE-180, ECa-109, KYSE-510, KYSE-520, KYSE-410, 108Ca, TE-1, EC18 and HKESC-1) (**A,B**) and in eight pairs of matched ESCC and noncancerous tissues (**C,D**), with GAPDH as a loading control in both panels. (**E**) Increased GOLPH3 expression in eight pairs of matched ESCC and the noncancerous tissues was further verified by immunohistochemistry. ANT, adjacent noncancerous tissue; ESCC, esophageal squamous cell cancer; GAPDH, glyceraldehyde 3-phosphate dehydrogenase; GOLPH3, Golgi phosphoprotein 3; NEEC, normal human esophageal epithelial cells; T, ESCC tissue.

qRT-PCR and western blotting analyses were respectively conducted to determine the levels of GOLPH3 mRNA and protein, in both normal human esophageal epithelial cells and ESCC cell lines, including KYSE-30, KYSE-140, KYSE-180, ECa-109, KYSE-510, KYSE-520, KYSE-410, 108Ca, TE-1, EC18, and HKESC-1. GOLPH3 expression showed barely detectable in normal human esophageal cells, whereas a notably higher level of GOLPH3 expression was found in all tumor cell lines at both the mRNA and protein levels (Figure 1A, 1B). Furthermore, comparative analysis of GOLPH3 expression was conducted on eight pairs of matched ESCC tissue and adjacent noncancerous tissue. The expression of GOLPH3 in all eight ESCC samples was much higher than the paired adjacent noncancerous tissue (Figure 1C). Similarly, the expression level of GOLPH3 mRNA was also increased in ESCCs compared with that in the adjacent nonmalignant esophageal tissues (Figure 1D). This finding was further verified by IHC in the eight paired ESCC and noncancerous tissue samples (Figure 1E).

### Association between increased expression of GOLPH3 and progression of ESCC

To further examine whether high expression of GOLPH3 protein is linked to the clinical progression of ESCC, the following samples were subjected to IHC staining with a human GOLPH3 antibody: 155 paraffin-embedded, archived ESCC tissue samples, including 9 cases of stage I, 64 cases of stage IIA, 14 cases of stage IIB, 54 cases of stage III and 14 cases of stage IV tumors, and 10 normal esophageal tissue samples. The mean age of the 155 ESCC patients was 57 years (range 35 to 82 years), and follow-up data was available for all patients. The duration of follow-up ranged from 1 to 77 months, with a median follow-up period of 32.17 months. A total of 91 deaths were reported during follow-up.

**Figure 2:**
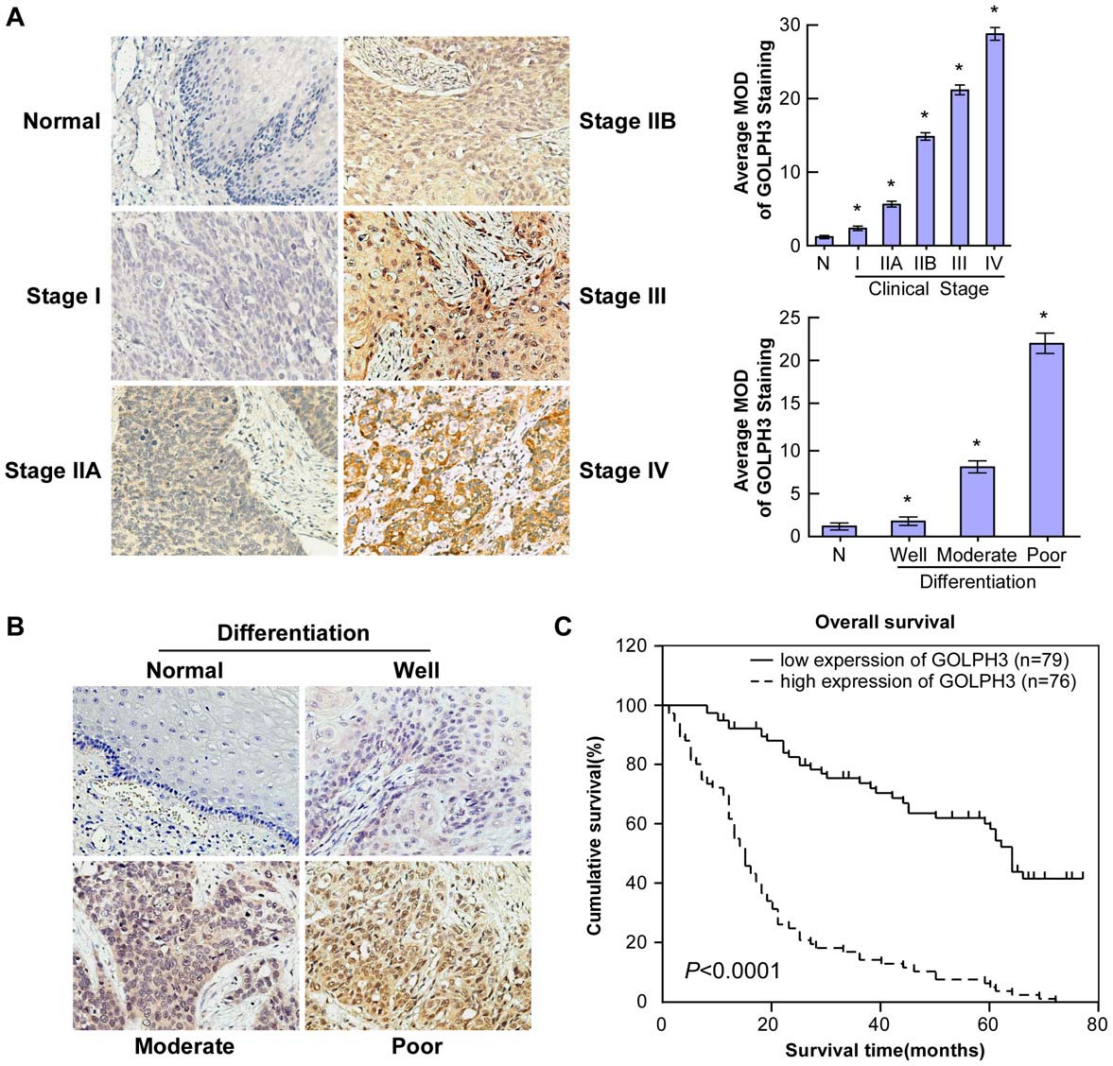
The expression level of GOLPH3 corresponded with the progression of ESCC. Average expression of GOLPH3 increased progressively from stage I to stage IV tumors, and was significantly higher than that in normal esophageal tissues (**A**). Moreover, GOLPH3 expression in poorly differentiated ESCC tissues was significantly higher than the expression in ESCC tissues that were moderately or well differentiated (**B**). Kaplan–Meier curves with univariable analyses (log-rank) for patients with low GOLPH3 expression (bold line) versus high GOLPH3 expression (dotted line) tumors (**C**). N, normal esophageal tissues; MOD, mean optical density.

The results of IHC staining are summarized in Table 1. GOLPH3 protein was highly expressed in 76 of 155 (49.0%) human ESCC samples. In contrast, only minimal levels of GOLPH3 were detected in normal esophageal tissues and adjacent noncancerous tissues (Figure 1E, 2A, 2B). Furthermore, the expression level of GOLPH3 correlated with the progression of ESCC (Figure 2A). The average expression level of GOLPH3 increased progressively through tumor stages I to IV, and levels were significantly higher than those in normal esophageal tissue. Moreover, GOLPH3 expression in ESCC tissues with poor differentiation was statistically significantly higher than that in well or moderately differentiated ESCC tissues (Figure 2B). Statistical analyses showed no relationship between patient gender or age and the expression level of GOLPH3. However, GOLPH3 expression was strongly associated with clinical stage (*P*<0.0001), T classification (*P*<0.0001), N classification (*P*<0.0001), M classification (*P* = 0.004) and histological differentiation (*P* = 0.001) (Table 2). Spearman analysis also revealed a correlation between GOLPH3 expression level and the clinical stage (r = 0.591, *P*<0.0001), T classification (r = 0.336, *P*<0.0001), N classification (r = 0.540, *P*<0.0001), M classification (r = 0.231, *P* = 0.004) and histological differentiation (r = 0.294, *P*<0.0001) (Table 3). Taken together, these observations support the hypothesis that the progression of ESCC is associated with increased GOLPH3 expression.

**Table 2:** Correlation between GOLPH3 expression and clinicopathologic characteristics of ESCC.

Characteristics		GOLPH3		*P* value
		Low-expression	High-expression	
		No. cases (%)	No. cases (%)	
**Gender**	Male	59(74.7)	59(77.6)	0.667
	Female	20(25.3)	17(22.4)	
**Age (years)**	≤57	40(50.6)	43(56.6)	0.458
	>57	39(49.4)	33(43.4)	
**Clinical stage**	I	9(11.4)	0(0.0)	**<0.0001**
	IIA	51(64.6)	13(17.1)	
	IIB	5(6.3)	9(11.8)	
	III	12(15.2)	42(55.3)	
	IV	2(2.5)	12(15.8)	
**T classification**	T1	10(12.7)	1(1.3)	**<0.0001**
	T2	28(35.4)	15(19.7)	
	T3	40(50.6)	51(67.1)	
	T4	1(1.3)	9(11.8)	
**N classification**	N0	61(77.2)	17(22.4)	**<0.0001**
	N1	17(21.5)	57(75.0)	
	N2	1(1.3)	2(2.6)	
**M classification**	M0	77(97.5)	64(84.2)	**0.004**
	M1	2(2.5)	12(15.8)	
**Differentiation**	Well	31(39.2)	16(21.1)	**0.001**
	Moderate	33(41.8)	24(31.6)	
	Poor	15(19.0)	36(47.4)	
**Vital status**	Alive	43(54.4)	21(27.6)	**0.001**
	Tumor-related death	36(45.6)	53(69.7)	
	Tumor-unrelated death	0(0.0)	2(2.6)	

**Table 3:** Spearman correlation analysis between GOLPH3 expression and clinical pathologic factors.

Variables	GOLPH3 expression level	
	Spearman correlation	*P*-value
**Age**	−0.062	0.446
**Clinical stage**	0.591	<0.0001
**T classification**	0.336	<0.0001
**N classification**	0.540	<0.0001
**M classification**	0.231	0.004
**Differentiation**	0.294	<0.0001
**Survival**	0.282	<0.0001

### Association between GOLPH3 expression and prognosis, and survival rates for ESCC patients

Statistical analysis revealed an inverse correlation between the expression level of GOLPH3 and the patient's vital status (*P* = 0.001; Table 2). While the Spearman analysis also revealed similar relations (r = 0.282, *P*<0.0001; Table 3). Furthermore, a log-rank test and Kaplan-Meier analysis were used to calculate the effect of GOLPH3 on survival. The log-rank test showed that the expression level of GOLPH3 protein attested remarkably to patients' survival time (*P*<0.0001; Figure 2C). More specifically, the median survival time of patients with high expression levels of GOLPH3 protein was only 15 months, whereas the median survival time of those with low levels of GOLPH3 was 45 months. The cumulative 5-year survival rate was 40.51% (95%CI, 0.2944–0.5157) in the low GOLPH3 expression group (n = 79), whereas in the high GOLPH3 expression group (n = 76; Figure 2C), the survival rate was only 6.58% (95% CI, 0.0088–0.1228).

Clinical stage, GOLPH3 expression, TNM classification, histological differentiation, tumor location, therapy, completeness of surgical resection and complications were analyzed using univariable and multivariable Cox regression analyses. Univariable analyses revealed that clinical stage, GOLPH3 expression, TNM classification, and histological differentiation were significant predictors of ESCC (Table 4). Multivariable analysis showed that clinical stage, GOLPH3 expression, TNM classification, and histological differentiation were independent predictors for ESCC on the basis of changes in likelihood interactions between the parameters listed in univariable regression analyses (*P*<0.05; Table 4).

**Table 4:** Univariable and multivariable analyses of various prognostic parameters in patients with ESCC.

	Univariable analysis		Multivariable analysis		
	P value	Regression coefficient (SE)	P value	Relative	95% confidence interval
				risk	
**Clinical stage**	<0.001	0.365(0.089)	0.017	0.543	0.330–0.895
**Expression of GOLPH3**	<0.001	1.417 (0.226)	<0.001	3.344	1.816–6.157
**T classification**	<0.001	0.536(0.153)	0.033	1.567	1.037–2.369
**N classification**	<0.001	0.811(0.200)	0.004	3.276	1.467–7.314
**M classification**	0.009	0.819(0.312)	0.043	2.312	1.027–5.206
**Differentiation**	<0.001	0.582(0.141)	0.025	1.434	1.046–1.964
**Location**	0.658	0.076(0.173)	0.617	1.088	0.781–1.516
**Therapy**	0.485	−0.217(0.311)	0.521	0.802	0.409–1.573
**Completeness of surgical resection**	0.218	−0.726(0.589)	0.233	0.479	0.143–1.606
**Complication**	0.713	−0.096(0.26)	0.909	1.033	0.596–1.789

**Figure 3:**
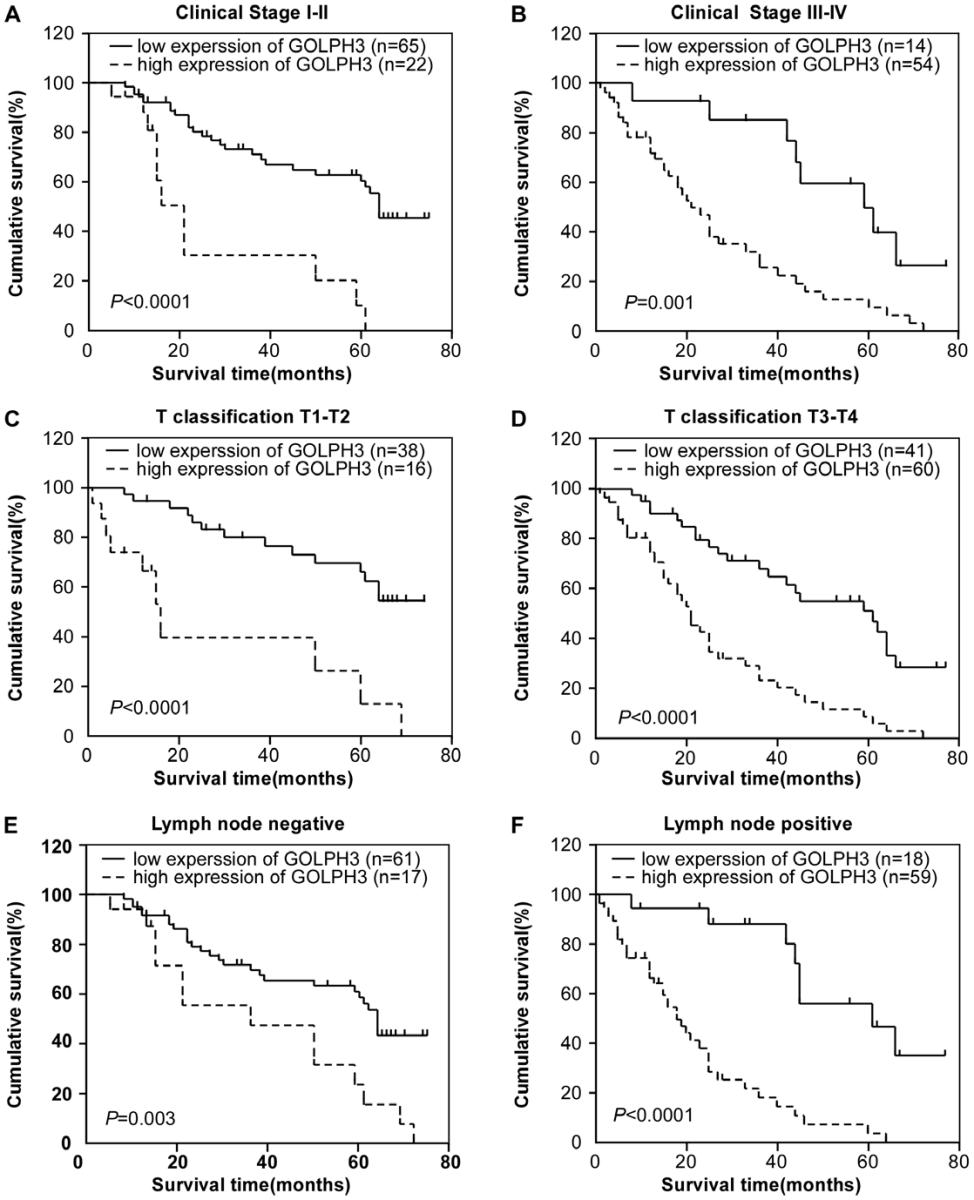
Kaplan–Meier analysis showing the overall survival of ESCC patients categorized according to the clinical stage, T classification, lymph node metastasis and status of GOLPH3 expression. Statistical significance of the difference between curves of GOLPH3 high-expressing and low-expressing patients was compared in clinical stage I to II (A) and clinical stage III to IV (B) patient subgroups, T classification T1 to T2 (C) and T classification T3 to T4 (D) patient subgroups, lymph node metastasis negative (E) and lymph node metastasis positive (F) patient subgroups.

We further examined the prognostic value of GOLPH3 expression in different subgroups of ESCC patients stratified according to the clinical stage, T classification, and lymph node metastasis. Significant correlation between high GOLPH3 expression and shorter overall survival time was found. Individuals with high GOLPH3 expression had significantly shorter overall survival than those with low expression in both the stage I+II subgroup (n = 87, *P*<0.0001; Figure 3A) and the stage III+IV subgroup (n = 68, *P* = 0.001; Figure 3B), indicating that GOLPH3 could be a valuable prognostic marker for ESCC in all disease stages. Similarly, the overall survival was significantly shorter in patients with high GOLPH3 expression in both the T1+T2 subgroup (n = 54, *P*<0.0001; Figure 3C) and the T3+T4 subgroup (n = 101, *P*<0.0001; Figure 3D), or in lymph node metastasis negative (n = 78, *P* = 0.003; Figure 3E) and positive patients (n = 77, *P*<0.0001; Figure 3F).

## Discussion

A recapitulation of our results: firstly, a notably higher level of GOLPH3 expression was present in all tumor cell lines at both mRNA and protein levels, while bare expression of GOLPH3 was detected in normal human esophageal cells. Secondly, a higher level of GOLPH3 expression was observed in all eight human primary ESCC samples compared with paired adjacent noncancerous tissue. Thirdly, results of IHC staining showed high expression of the GOLPH3 protein in 49.0% of the examined ESCC samples, but minor expression in normal and adjacent noncancerous tissues.

GOLPH3 is a phosphorylated protein that localizes to the Golgi apparatus, interacts with the cytoskeleton, and is involved in the maintenance of the Golgi [11]. GOLPH3 is associated with vacuolar protein sorting-associated protein 35 (Vps35), a component of the retromer complex, which is responsible for retrograde transport of certain cell surface receptors and other cargo proteins from endosomes to the *trans*-Golgi network. Furthermore, deletion of *Vps35* in budding yeast leads to rapamycin hypersensitivity, consistent with an impairment of TORC1 signaling [6]. High GOLPH3 expression has been found to correlate with hyperactivation of mTORC2 and mTORC1 signaling in human cells. In xenograft experiments conducted in immunodeficient mice, tumor cells with overexpressed GOLPH3 showed increased sensitivity to therapy with the TORC1 inhibitor, rapamycin [6], [11]–[12]. These results suggest that GOLPH3-dependent oncogenesis is dependent on mTORC signaling, and is therefore sensitive to mTOR inhibitors in these preclinical models [11]–[12], [16]–[17]. Recently, *GOLPH3* has been demonstrated to be a potential novel oncogene that is involved in vesicular trafficking. The results of gain- and loss-of-function studies *in vitro* and *in vivo* have validated *GOLPH3* as a potent oncogene. The *GOLPH3* gene is located on the human chromosome 5p13 and is frequently amplified in several solid tumor types, such as cancer of the lung, ovary, breast, prostate, and skin (melanoma) [6]. Human rhabdomyosarcoma cell lines and biopsy specimens exhibited an increased expression of both GOLPH3 and GOLPH3-like (GOLPH3L) mRNA and protein. In addition, GOLPH3 and GOLPH3L knockdown by small interfering RNA prevented the proliferation of human rhabdomyosarcoma cell lines [7].

Current experimental and clinical evidence indicating GOLPH3's involvement in human tumors is limited. In a pilot study, increased expression of GOLPH3 was found in more than half of patients with glioma, and the level of GOLPH3 expression was associated with tumor severity [8]. Using long serial analysis of gene expression, GOLPH3 was identified as a novel androgen-responsive gene in prostate cancer [9]. In consistence with these studies, our results reveal that high GOLPH3 expression is an independent prognostic factor of ESCC patients. High GOLPH3 expression strongly associates with clinical stage, TNM classification, and histological differentiation, which indicates that increased GOLPH3 expression is associated with the progression of ESCC. Moreover, we studied the relationship between GOLPH3 expression and patient prognosis, revealing that the high expression level of GOLPH3 protein in ESCC corresponds remarkably with patients' survival time. Finally, stratified analysis exhibited that significant correlation between high GOLPH3 expression and shorter overall survival time was found at all disease stages, TNM classification, and lymph node metastasis subgroups of ESCC, indicating that GOLPH3 could be a valuable biomarker for prediction of the severity of ESCC and the prognosis for ESCC patients. Our results suggest that the high expression of GOLPH3 may play an important role in the development and progression of ESCC tumorigenesis, although its exact mechanisms remain for future exploration. GOLPH3 may participate in the ESCC tumorigenesis as a result of its stimulative effect on mTOR. Further studies plan on exploring the function of GOLPH3 and the mechanism for its up-regulation in ESCC tumor, and to clarify whether GOLPH3 modulates mTOR signalling and rapamycin sensitivity in ESCC.

In conclusion, our study demonstrates that a substantially higher level of GOLPH3 expression in ESCC exists within cell lines and tissues at both mRNA and protein levels. A high expression of GOLPH3 in ESCC patients is positively associated with advanced clinical stage, high histological grade, and higher TNM stage. ESCC patients expressing high levels of GOLPH3 also exhibit a substantially lower 5-year overall survival rate than GOLPH3-low expression patients. Thus, our study provides evidence that GOLPH3 may play an important role in the development, differentiation, and carcinogenesis of ESCC, and therefore, could be utilized in diagnosis and as a prognostic indicator in ESCC patients.

## Supporting Information

Figure S1 **Distribution of immunohistochemistry score of all cases by differentiation.**
(TIF)

Figure S2 **Distribution of immunohistochemistry score of all cases by clinical stage.**
(TIF)

Table S1 **Immunohistochemistry score distribution of all cases (by Clinical stage and differentiation).**
(DOC)
