# Supplementary figures and images for: High Expression of GOLPH3 in Esophageal Squamous Cell Carcinoma Correlates with Poor Prognosis
Source: PLoS One. 2012 Oct 2;7(10):e45622. doi: 10.1371/journal.pone.0045622 (PMC3462781; doi:10.1371/journal.pone.0045622)

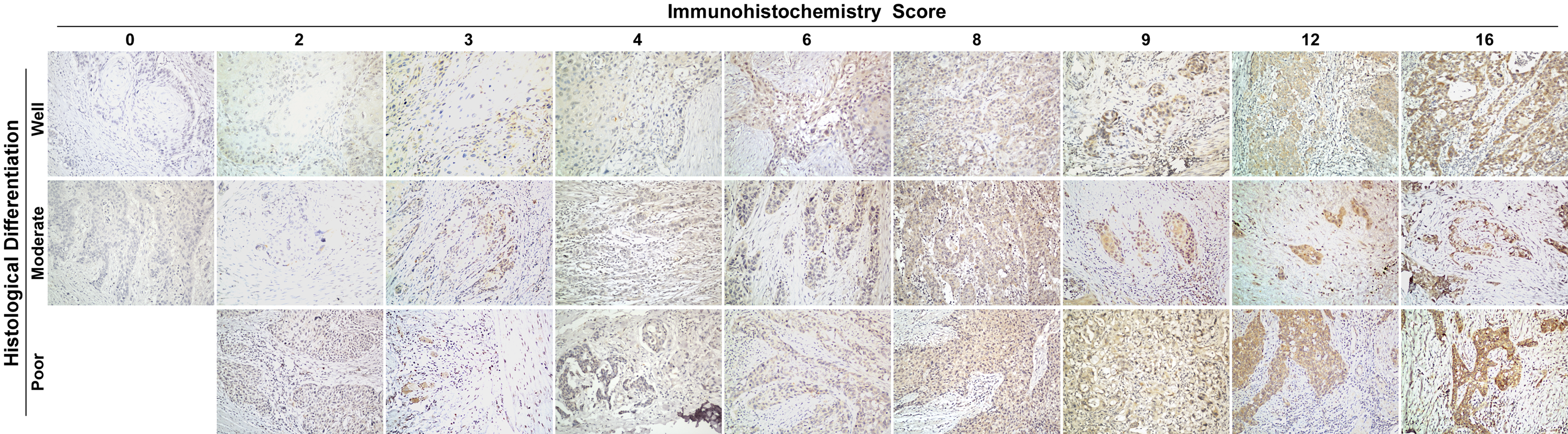

Supplement: Figure S1 — Distribution of immunohistochemistry score of all cases by differentiation. (TIF) [file pone.0045622.s001.tif]

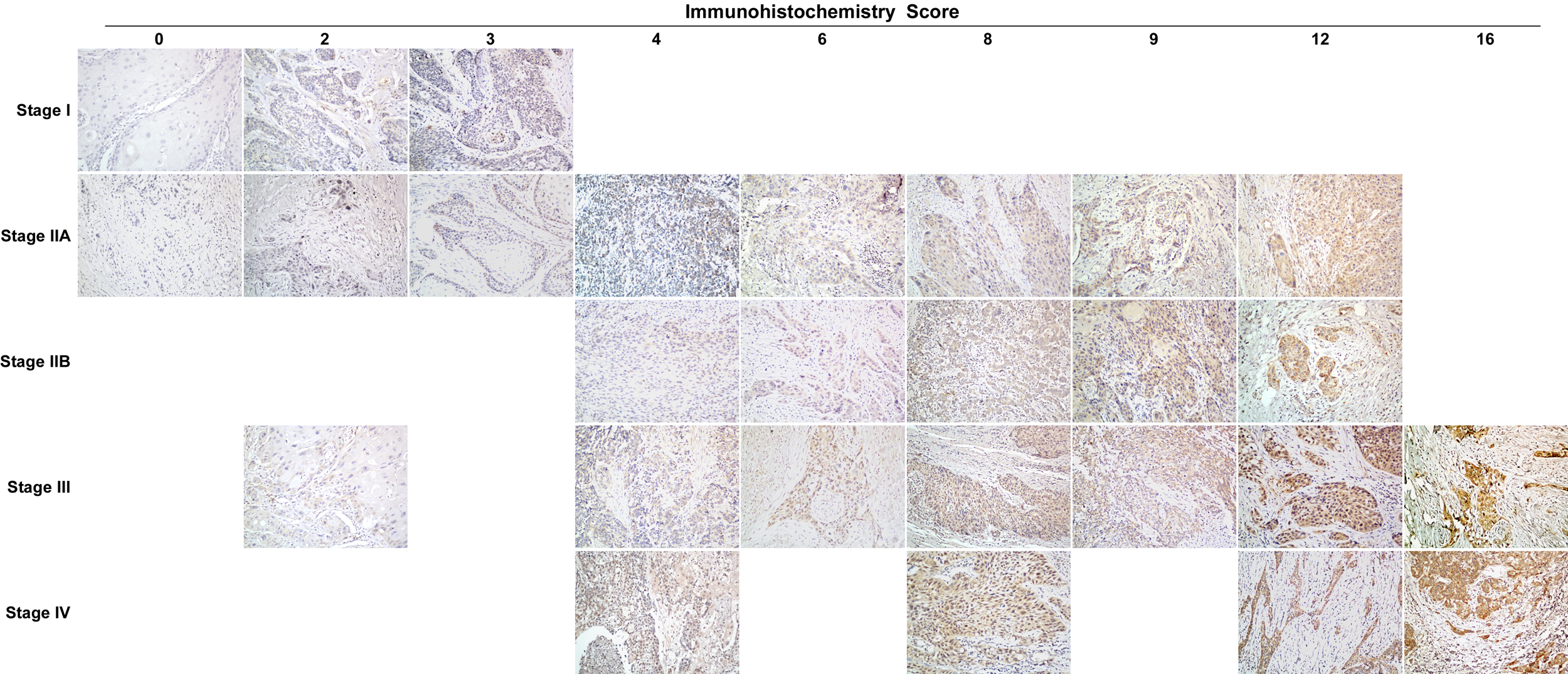

Supplement: Figure S2 — Distribution of immunohistochemistry score of all cases by clinical stage. (TIF) [file pone.0045622.s002.tif]
