# Supplementary material for: High Expression of GOLPH3 in Esophageal Squamous Cell Carcinoma Correlates with Poor Prognosis
Source: PLoS One. 2012 Oct 2;7(10):e45622. doi: 10.1371/journal.pone.0045622 (PMC3462781; doi:10.1371/journal.pone.0045622)
Supplement: Table S1 — Immunohistochemistry score distribution of all cases (by Clinical stage and differentiation). (DOC) [file pone.0045622.s003.doc]

**Table S1. Immunohistochemistry score distribution of all cases (by Clinical stage and differentiation)**

| **Immunohistochemistry**  **score** | **Number of cases (%)** | | | | | | | |
| --- | --- | --- | --- | --- | --- | --- | --- | --- |
|  | **Clinical Stage** | | | | | **Histological Differentiation** | | |
|  | **I** | **IIA** | **IIB** | **III** | **IV** | **Well** | **Moderate** | **Poor** |
| **0** | 3(33.3) | 2(3.1) | 0(0.0) | 0(0.0) | 0(0.0) | 2(4.3) | 3(5.3) | 0(0.0) |
| **1** | 0(0.0) | 0(0.0) | 0(0.0) | 0(0.0) | 0(0.0) | 0(0.0) | 0(0.0) | 0(0.0) |
| **2** | 2(22.2) | 10(15.6) | 0(0.0) | 1(1.9) | 0(0.0) | 4(8.5) | 6(10.5) | 3(5.9) |
| **3** | 4(44.4) | 12(18.8) | 0(0.0) | 0(0.0) | 0(0.0) | 4(8.5) | 6(10.5) | 6(11.8) |
| **4** | 0(0.0) | 19(29.7) | 2(14.3) | 8(14.8) | 2(14.3) | 17（36.2） | 10(17.5) | 4(7.8) |
| **6** | 0(0.0) | 9(14.1) | 2(14.3) | 1(1.9) | 0(0.0) | 4(8.5) | 7(12.3) | 1(2.0) |
| **8** | 0(0.0) | 8(12.5) | 7(50.0) | 13(24.1) | 1(7.1) | 8(17.0) | 16(28.1) | 5(9.8) |
| **9** | 0(0.0) | 2(3.1) | 2(14.3) | 14(25.9) | 0(0.0) | 3(6.4) | 4(7.0) | 11(21.6) |
| **12** | 0(0.0) | 2(3.1) | 1(7.1) | 12(22.2) | 9(64.3) | 4(8.5) | 3(5.3) | 17(33.3) |
| **16** | 0(0.0) | 0(0.0) | 0(0.0) | 5(9.3) | 2(14.3) | 1(2.1) | 2(3.5) | 4(7.8) |
| **Total** | 9 | 64 | 14 | 54 | 14 | 47 | 57 | 51 |
| **Mean** | 6.86 |  |  |  |  |  |  |  |
| **Median** | 8 |  |  |  |  |  |  |  |
|  |  | | | | | | | |
